# Pancreatic cancer-associated myofibroblasts: a review

**DOI:** 10.7150/ijbs.121234

**Published:** 2026-02-18

**Authors:** Marcin Banacki, Iwona Inkielewicz-Stepniak

**Affiliations:** Department of Pharmaceutical Pathophysiology, Medical University of Gdańsk, Gdańsk, Poland.

**Keywords:** Pancreatic cancer, myofibroblast, fibrosis, microenvironment, stellate cell, extracellular matrix

## Abstract

Pancreatic cancer is a very deadly disease, with no effective therapy currently employed in clinical practice, highlighting the urgent need for new therapeutic strategies. Cancer cells represent a minority within the pancreatic tumor, which is characterized by a pronounced stromal compartment. As fibrosis is characteristic of pancreatic ductal adenocarcinoma, the cell population of particular interest is the pancreatic cancer-associated myofibroblast population, observed to be the main extracellular matrix producers. Recent studies revealed a plurality of myofibroblast functions in the pancreatic tumor, beyond their role in matrix secretion: they were noted to promote cancer cell proliferation and invasiveness in *in vitro* and *in vivo* studies, and have been described as potential prognostic biomarkers, along with stromal collagen content. Moreover, myofibroblasts were found in precancerous pancreatic lesions, and may thus be involved in pancreatic carcinogenesis. Paradoxically, depletion of myofibroblasts had detrimental effects on the outcome in an *in vivo* study, and their precise role in the disease remains unclear. This review summarizes for the first time studies on pancreatic cancer-associated myofibroblasts, focusing on the origin, function, biomarker potential, and heterogeneity of these cells in the pancreatic tumor, aiming to elucidate their role in pancreatic cancer progression.

## Background

The cellular portion of the tumor microenvironment consists of cells belonging to a number of cell types beyond cancer cells, including various immune cells, endothelial cells, and fibroblasts 1. These cells perform a plurality of functions within the tumor, inducing (or inhibiting) angiogenesis, immune tolerance or cancer cell proliferation 1. Microenvironment cells are also responsible for the secretion of extracellular matrix (ECM) proteins, which form the acellular portion of the microenvironment 1. This phenomenon, dubbed “desmoplastic reaction”, furthers the pro-tumor effects of the microenvironment, inducing angiogenesis 2 or serving as a nutritional source for cancer cells 3. Cancer-associated fibroblasts are known as primary producers of ECM in tumors 4.

Pancreatic adenocarcinoma is a yet-unbeatable disease, a characteristic of which is its low 5-year survival rate 5. Dense stroma is a characteristic of the pancreatic tumor 6, decreasing drug penetration and thus, diminishing therapy effectiveness 7. As such, fibroblasts present within the tumor's microenvironment are of interest from both the clinical and biological perspective. Emerging evidence points to the presence of three main fibroblast types associated are distinguished in this disease: inflammatory cancer-associated fibroblasts, which are characterized by their low expression of alpha-smooth muscle actin (αSMA), secrete proinflammatory cytokines, such as IL-6 8; antigen-presenting cancer-associated fibroblasts, which express HLA-II-group antigen-presenting molecules 9, and myofibroblasts, which produce the ECM constituents collagen I and hyaluronan, and are characterized by their high expression of alpha-smooth muscle actin 10,11. Thus, myofibroblasts are a fibroblast population of crucial importance for the course of pancreatic cancer. The novelty of that classification leads to an unclear nomenclature used to describe fibroblasts in the tumor environment, as the name “myofibroblasts” was sometimes used to describe the overall cancer-associated fibroblast population in the tumor 12. The different functions of cancer-associated fibroblast population in the pancreatic tumor warrant the application of the name “myofibroblasts” as strictly αSMA-high, ECM-producing cells with no interleukin signalling activation of the pancreatic adenocarcinoma tumor microenvironment, and this term is used in such this meaning in this review.

Despite the myofibroblast's role in desmoplastic reaction, depletion of these cells in the tumor also worsened the course of the disease 13. Nevertheless, new studies show that the pro-cancer role of these cells is not limited to ECM formation, extending to enhancing cancer cell chemoresistance or proliferation, among others. Furthermore, myofibroblasts dominated the microenvironment of pre-cancerous, high-grade pancreatic lesions, suggesting a role in carcinogenesis 14. This review summarizes current state of literature regarding cancer-associated myofibroblasts, and attempts to elucidate the source of the apparent ambiguity of the myofibroblasts' role in the pancreatic tumor microenvironment, in addition to describing their origins and heterogeneity.

## Origins of pancreatic adenocarcinoma-associated myofibroblasts

### Sources of pancreatic adenocarcinoma-associated myofibroblasts

The origins of myofibroblasts in pancreatic tumor microenvironment are currently unclear. There is evidence for pancreatic stellate cells forming myofibroblasts, myofibroblastic conversion of the *in situ* fibroblasts and for the bone marrow origin of myofibroblasts in the course of pancreatic cancer.

The pancreas contains fibroblast-like stellate cells, characterized by their high expression of vimentin, adipoliphilin and cytoglobin, and retinoid droplets content 15. Under physiological conditions, these cells are quiescent and possibly exhibit only limited ECM remodelling functions, as they secrete metalloproteinases and their inhibitors 16. However, growth factors or cytokines - such as may be released from the pancreatic tumor 17 - may activate them and subsequently decrease vimentin, cause loss of retinoid content in addition to triggering a large increase in αSMA expression 10,18,19. Activated pancreatic stellate cells are able to proliferate 6, in addition to secreting ECM components such as collagens (type I, type III), laminin and fibronectin 10. These traits, together with the aforementioned αSMA production, are characteristic to myofibroblasts 10. A lineage-tracing study established that in pancreatic adenocarcinoma, stellate cell-derived myofibroblasts represent only a minor fraction (10% - 15%) of cancer-associated fibroblasts 20.

Bone marrow appears to also be a source of pancreatic stellate cells in mice, as found by Watanabe et al., who observed that 8 weeks post-transplantation, 8.7% of pancreatic stellate cells were donor-derived, and that induction of pancreatic fibrosis (concomitant with stellate cell activation) has resulted in that percentage being increased to over 20% 21. Thus, bone marrow-derived cells may be concluded to be a source of stellate cells, which may transition into myofibroblasts during inflammation, and inflammation itself increases the amount of bone marrow-derived cells in the pancreas. While neither of these studies have tested pancreatic adenocarcinoma tumor, inflammation is directly related to pancreatic carcinogenesis 22.

There is a possibility of pancreatic fibroblast recruitment into the tumor and their subsequent activation. Fibroblasts were identified in the healthy human pancreas, differing from stellate cells by their lack of retinoid droplets. Medium conditioned by fibroblasts from an older person's pancreas was found to increase the growth rate of Panc10.05 and MIA-PaCa-2 pancreatic cancer cells, which was not observed when medium conditioned by a younger person's pancreatic fibroblasts was used 23. Interestingly, the isolated fibroblasts were observed to express αSMA, suggesting a possibility of a spontaneous fibroblast activation during cell culture 24,25. While that study shows that activated pancreatic fibroblasts exhibit a higher pro-tumor potential with aging, these cells may not be described as true myofibroblasts due to a lack of cancer cell-related signalling, essential for the development of myofibroblast phenotype 8, in the culture 23. As the isolated fibroblasts were noted to express Gli-1 23 - a transcription factor known to designate pancreatic fibroblast populations giving rise to pancreatic myofibroblasts 25 - these results may still be relevant for the role myofibroblasts in PDAC.

The adipose tissue is a known source of mesenchymal stem cells 26, which were observed to transform into the pancreatic cancer-associated myofibroblasts and inflammatory fibroblasts *in vitro* upon coculture with pancreatic cancer cells and later determined to secrete a cancer cell motility-enhancing ECM when exposed to pancreatic cancer cell-conditioned medium 26. Endothelial cells proximal to the tumor are also a known source of fibroblasts in various cancers, including PDAC 27-29, differentiating into fibroblasts upon stimulation with TNF-α 29. These endothelial-derived fibroblastic cells were noted to have ECM-secretory capabilities 29, proving that they may form a part of the myofibroblastic subpopulation of CAFs in PDAC.

Gao, Li, Cheng et al. conducted a single-cell study of several different malignant tumors, including pancreatic cancer tumors. They have established the pericyte or smooth muscle cell origin of the terminally-differentiated myofibroblasts, noting an abundance of likely pericyte-derived myofibroblasts in pancreatic tumors; the less-differentiated myofibroblasts were described as originating from other cell types, predominantly normal fibroblasts 30.

### Molecular pathways and spatial signalling driving myofibroblast differentiation in PDAC

Various molecular pathways appear to have been implicated in the fibroblast-to-myofibroblast transition. TGFβ, produced by many different types of cells in the pancreatic tumor microenvironment, including pancreatic stellate cells, cancer cells and immune cells 30, is a known inducer of differentiation of fibroblasts to myofibroblasts 31,32. While tumor αSMA and collagen levels decrease upon inhibition of fibroblast TGFβ receptor in a mouse model study 32, it was found that such an inhibition decreases the overall tumor fibroblast count and does not deplete myofibroblasts 33. The proportion of myofibroblasts within the cancer microenvironment was observed to be further regulated by IL-1 signalling, which drives the differentiation of cancer-associated fibroblasts to inflammatory fibroblasts, underscoring the importance of interplay between IL-1 and TGFβ signalling in cancer-associated fibroblast differentiation 32. An important signal transducer in TGFβ-driven fibroblast-to-myofibroblast transition appears to be the NOX4 reactive oxygen species-generating activity induced by TGFβ: the reactive oxygen species cause DNA damage, which in turn activates the ATM kinase 34-36. Blocking reactive oxygen species production by treatment with an anti-oxidant or NOX4 inhibitor after TGFβ treatment was found to decrease the number of myofibroblasts within the tumor and prevent cancer associated fibroblasts from transitioning to myofibroblasts 37. Similarly, inhibiting ATM concurrently to TGFβ treatment was similarly observed to inhibit differentiation of cancer-associated fibroblasts to myofibroblasts and to somewhat revert myofibroblasts back to fibroblast phenotype, evident by the decrease in αSMA, collagen I and fibronectin expression in the cells 35. Importantly, it has been reported that the ATM can also transduce reactive oxygen species-related signal independently of DNA damage 36. However, no study of whether reactive oxygen species-related DNA damage is required for ATM activation and subsequent DNA damage has been conducted.

There is evidence for PI3K/Akt/MAPK pathway involvement in pancreatic stellate cell activation to myofibroblasts, as decreasing the PI3K inhibitor *PTEN* expression increased collagen synthesis and motility of these cells, while increasing *PTEN* expression increased stellate cell apoptosis rate 38. Moreover, platelet-derived growth factor (PDGF) is established to be involved in fibroblast activation in PDAC 38, inducing their proliferation by activating the PI3K/AKT pathway 39, further proving its importance in myofibroblast biology.

The Smoothened receptor signalling was also found to be overexpressed by isolated human pancreatic cancer-associated myofibroblasts (but not by normal pancreatic fibroblasts); the activation of this receptor resulted in an increase in Gli-1, reversed upon knockdown of Smoothened's gene 40. Inhibition of Smoothened has resulted in a decreased tumor growth and myofibroblast number coupled with an increased number of inflammatory cancer-associated fibroblasts in an animal model study of pancreatic cancer 41.

Protein kinase N2 is also thought to be important for the activation of stellate cells and their transformation to myofibroblasts: in mouse pancreatic stellate cells, deletion of the *PKN2* gene, coding for said kinase, resulted in an increased lipid storage by the stellate cells and a decreased number of αSMA fibres in the cells, apparent even after their stimulation with tumor-growth factor 1 (TGFβ1) 42, proving that the signalling from this kinase is important for maintenance of myofibroblast identity. However, the authors did not observe a decreased tumor collagen level in an orthotopic animal model bearing a knockout-*PKN2*. It appears that the ECM-secretory aspect of myofibroblasts is controlled independently of this kinase through the mTOR/4E-BP1 pathway, as its inhibition with a somatostatin analogue resulted in reduction of fibrosis and subsequent sensitization of the tumor to gemcitabine 43.

Sun et al. reported a significant role of tumor-associated macrophages in pancreatic cancer-associated myofibroblast formation: the cytokine IL-33 is secreted by inflammatory fibroblast population, activating the ST2 receptor on the macrophages. Signal is transduced further by ERK and MYC, stimulating the macrophages to produce CXCL3. This in turn subsequently activates the fibroblast CXCR2 receptor, triggering the fibroblast-to-myofibroblast transition; inhibiting the ST2 receptor has decreased the level of αSMA in tumor tissues 44.

A 2017 coculture study suggested that direct contact between cancer cells and stellate cells promoted differentiation of fibroblasts into myofibroblasts 8. However, subsequent work demonstrated that this contact is not strictly required; rather, proximity to cancer cells and exposure to short-range paracrine cues, particularly TGFβ, are sufficient to induce the myofibroblast phenotype. In contrast, fibroblastic cells located farther from tumour cells experience IL-1α/TGFβ-driven activation of the JAK/STAT pathway, giving rise to inflammatory cancer-associated fibroblasts that secrete cytokines such as IL-6 and CXCLs 32. The importance of spatial signalling molecule gradients was corroborated by a coculture study of adipose-derived stem cells (which transformed into fibroblasts) and pancreatic cancer cells 26. Thus, spatial organization and competing TGFβ versus IL-1/JAK/STAT signals determine CAF subtype specification.

## Heterogeneity

Recent research established that not only are there various populations of fibroblasts found within the pancreatic tumor, but there is also a considerable heterogeneity within the pancreatic cancer-associated myofibroblasts. While different marker molecules were noted to distinguish different myofibroblast subpopulations, there is no single, unified classification of these cells into subtypes.

Two patient-derived myofibroblast cell lines were also examined for heterogeneity. It had been revealed that they varied by the expression of (myo)fibroblast markers, with one cell line expressing FAP and PDGFR-α at a considerably lower rate than another. The “expression-low” myofibroblasts-conditioned medium was observed to significantly increase proliferation of patient-derived pancreatic cancer cells, while the “expression-high” cells-conditioned medium did not influence cancer cell proliferation, but decreased their migration 45. This points to the possible distinction between the myofibroblast subpopulations in terms of their influence on pancreatic cancer cells. However, as only two patient-derived myofibroblast cell lines were studied, no firm conclusion may be drawn.

Mucciolo et al. have observed two distinct pancreatic cancer-associated myofibroblasts subpopulations, differing by the presence of CD90. The CD90^+^ myofibroblasts appear to be mainly responsible for ECM production, as they were observed to have an increased expression of ECM-related genes. They also expressed myofibroblast markers *Acta2* and *Col1a1* at a higher rate than CD90^-^ cells, which had a higher expression of EGFR signalling-related genes and of secreted, metastasis-promoting proteins, such as *Spp1* and *Sema3e* 51, which was consistent with the observation that these myofibroblasts drive metastasis. The notion that there are two subtypes of myofibroblasts, with one of them being the primary producer of ECM, may explain the mechanism behind the emergence of collagen-high stromal type in pancreatic cancer 49: possibly, a collagen-high ECM is formed due to there being more CD90^+^ myofibroblasts in the early part of carcinogenesis. Importantly, precise functions of these myofibroblast subpopulations were not determined; an animal model xenograft experiment using pancreatic cancer cells and either CD90^+^ or CD90^-^ myofibroblasts could be conducted to shed light on this subject.

A yet another subset of myofibroblasts are the senescent myofibroblasts which were observed to occur in the vicinity of tumor ducts. Their presence caused a shift of macrophage phenotype from the anti-cancer M1 to the pro-cancer M2 phenotype. Moreover, these myofibroblasts increase in number as the disease progresses, demonstrating a possible senescence induction by the tumor 50. Whether the presence of such myofibroblasts is a cause of disease progression, or the tumor cells induce senescence in myofibroblasts as the disease progresses is currently unknown.

## Functions

### Myofibroblasts in cancer cell regulation

**Figure 2:**
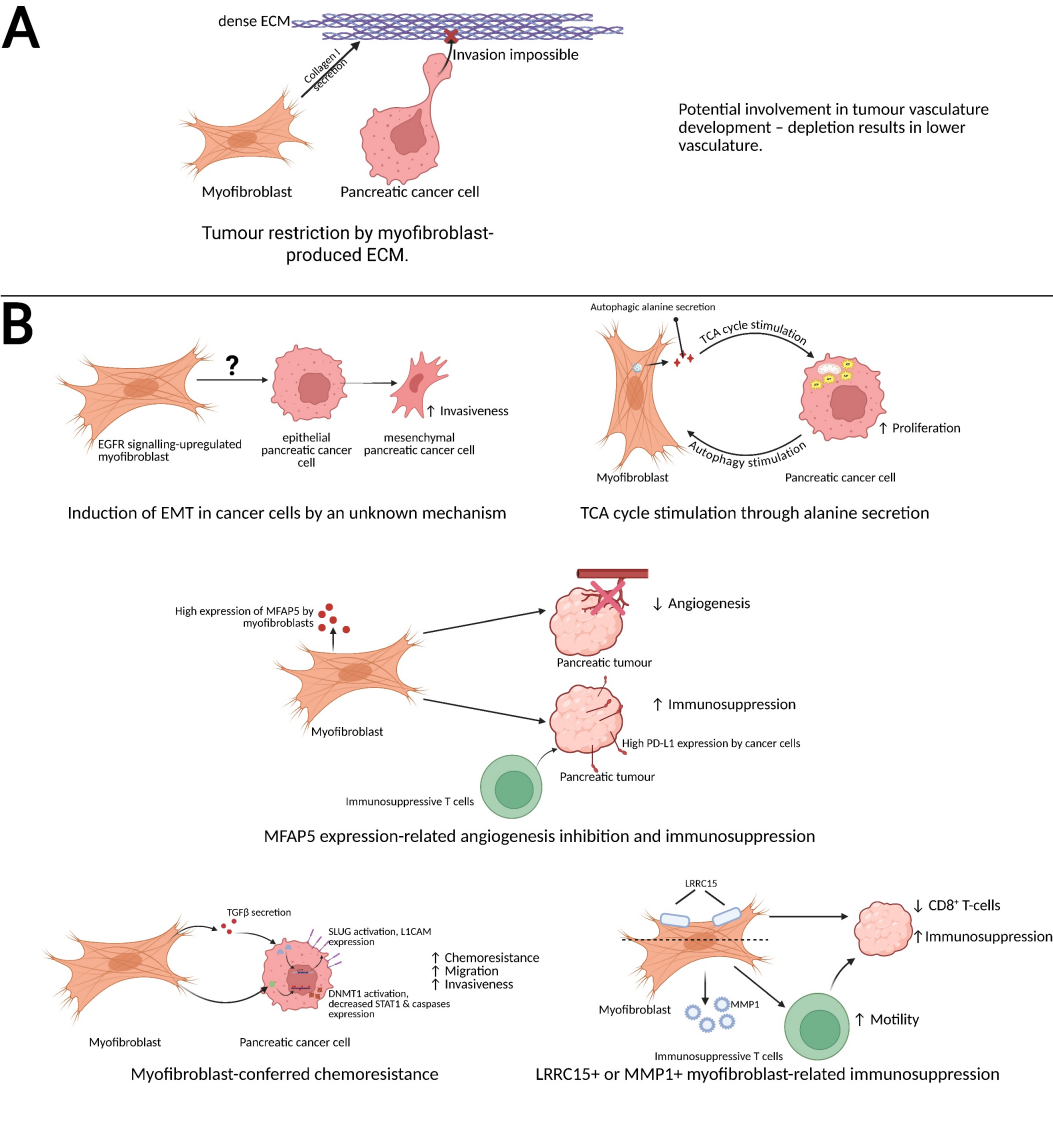
**Schematic representation of the anti-cancer and pro-cancer functions of pancreatic cancer-associated myofibroblasts.** (A) Myofibroblast-secreted collagen I serves as an obstacle for pancreatic cancer cell invasiveness 70 and potentially is involved in tumor vasculature development 13. (B, top left) EGFR-upregulated myofibroblasts induce the epithelial-mesenchymal transition of pancreatic cancer cells, increasing their invasiveness 51. (B, top right) Myofibroblasts secrete alanine, a product of their autophagic turnover, which stimulates the pancreatic cancer cell's TCA cycle, increasing energy production and proliferation rate 59. (B, centre) MFAP5-expressing myofibroblasts inhibit tumor angiogenesis create immunosuppressive microenvironment by inducing PDL-1 expression in cancer cells and causing immunosuppressive T cell recruitment 62. (B, bottom left) Myofibroblasts secrete TGFβ, which causes the cancer cell to express SLUG and L1CAM, and activate pancreatic cancer cell's DNMT1, which causes a decreased expression of STAT1 and caspases; these processes result in an increased migration rate, chemoresistance and invasiveness 57,58. (B, bottom right) Similarly, LRRC15^+^ myofibroblasts and MMP1^+^ myofibrobroblasts create an immunosuppressive microenvironment in PDAC 30,64. Created with BioRender.com

Myofibroblasts are thought to be responsible for a number of pancreatic cancer-related processes (**Figure 2**), including promoting tumor invasiveness or secreting the ECM. It is currently understood that pancreatic cancer-associated myofibroblasts also perform various other functions within the pancreatic tumor. A portion of myofibroblasts stimulated by TGFβ treatment was noted to secrete an EGFR ligand - amphiregulin - in an autocrine fashion. Such EGFR-activated myofibroblasts were observed to stimulate epithelial-mesenchymal transition (EMT) in cancer cells and increase their invasiveness in a mouse model of pancreatic cancer 51.

Conversely to the above, a mouse model study of pancreatic cancer using the human CAPAN-1 cell line injected alone or together with human myofibroblasts isolated from metastatic sites of pancreatic cancer demonstrated that the myofibroblasts confer an increase in invasiveness onto the cancer cells 46. Whether this effect would also be observed with myofibroblasts isolated from the primary tumor is unknown. Moreover, it has been demonstrated that myofibroblasts may transition into inflammatory cancer-associated fibroblasts and secrete pro-invasive cytokines, such as interleukin 6 8,47. Thus, it is impossible to ascribe the effects of myofibroblast co-injection as occurring solely due to the presence of myofibroblasts.

Cultured mouse stellate cells were also noted to express *Zeb1* 52, a transcription factor commonly understood to be the main regulator of the EMT 53. However, its function was demonstrated to be different in these cells, as it was observed regulate to fibroblast survival and proliferation. *Zeb1* knockdown in the stellate cells was observed to decrease collagen I, metalloproteinase 9, and αSMA genes expression levels in activated pancreatic stellate cells, similarly to *Zeb1* haploinsufficiency, which has also caused a decrease in ECM-related, but not in αSMA, gene expression, indicating the importance of *Zeb1* in pancreatic stellate cell activation and subsequent desmoplastic reaction. Notably, wild-type activated stellate cell-conditioned medium was more effective than *Zeb1*-haploinsufficient activated stellate cells-conditioned medium at promoting mouse pancreatic cancer cell migration and proliferation. Moreover, a higher activity of Ras in cells derived from mouse pancreatic tumors bearing mutated *KRAS* gene (a known oncogene, often implicated in pancreatic cancer carcinogenesis) was noted when they were cultured in a wild-type activated stellate cells-conditioned medium, and, to a lesser extent, in a *Zeb1*-haploinsufficient wild type conditioned medium 52. As *Zeb1*-haploinsufficient mice bearing pancreas-specific *KRAS* mutation developed pancreatic tumors much more slowly than mice with both alleles of *Zeb1* active, and the Ras GTPase requires an external activator 54, it was concluded that the *Zeb1* transcription factor controls the secretome of stellate cells-derived myofibroblasts in such a way that they promote disease progression by increasing the Ras activity of the pancreatic cancer cells with mutated* KRAS* 52. The results of this study should be interpreted cautiously, however, as no cancer cell-stellate cell coculture experiment was performed 52, and thus, no cancer cell-related signalling required for myofibroblast development 8 was present and the resultant activated stellate cells do not really represent myofibroblasts as claimed by the title. Importantly, hypoxia, a known characteristic of PDAC 55, was also noted to increase pancreatic stellate cells' pro-tumor functions by increasing cancer cell invasiveness by secreting connective tissue growth factor 56.

A coculture experiment of pancreatic cancer cells or normal pancreatic duct epithelial cells with myofibroblasts has revealed a decrease in the expression of STAT1, subsequently decreasing the expression of caspases through an increased methylation of the STAT1 promoter by DNMT1 57. Moreover, a mouse model study revealed that when the myofibroblasts and T3M4 pancreatic cancer cells were co-injected into mice, the developed tumor was found to be resistant to etoposide, in addition to exhibiting a decreased expression of caspases and a more pronounced stroma 57. While inhibition of DNMT1 expression was performed *in vitro* and determined to sensitise the cells to etoposide therapy, a similar experiment has not been performed in the animal experiment phase of the study. As such, the mechanism behind that myofibroblast-conferred chemoresistance is unknown; it may be hypothesised to be related to aforementioned STAT1 inhibition, but can also be effect of dense stroma formation in the co-injected tumors, which could decrease drug penetration 7. Schäfer et al. observed that coculturing the normal pancreatic duct cells with pancreatic myofibroblasts also increases cell migration, invasiveness and chemoresistance through induction of L1CAM expression in the cells via the action of myofibroblast-secreted TGFβ on pancreatic cell's Slug 58. Overall, myofibroblasts may be understood to drive the carcinogenesis of pancreatic duct cells through increasing their chemoresistance and invasiveness.

Another process through which myofibroblasts may support pancreatic cancer cells is alanine secretion. Activated pancreatic stellate cells cocultured with PDAC cells were noted to produce it via autophagy, stimulated by pancreatic cancer cells. In turn, secreted alanine was observed to stimulate proliferation of the cancer cells by stimulating TCA cycle after its conversion to pyruvate. This effect was also observed *in vivo*, with autophagy inhibition decreasing tumor growth rate and increasing survival in a mouse model of pancreatic cancer 59, underscoring the significance of autophagy within myofibroblasts for pancreatic tumor growth.

### Myofibroblasts in pancreatic tumor microenvironment remodelling

Myofibroblasts' main function is established to be the production of ECM components - collagens (I, III), laminin and fibronectin - during pancreatic damage, including pancreatic neoplasm 6,10. This phenomenon, dubbed *desmoplastic reaction*, is a major obstacle in pancreatic cancer treatment, as the dense stroma surrounding the tumor decreases the drug penetration into the tumor 60,62, Despite that, attempts at ameliorating the disease by either depleting pancreatic cancer-associated myofibroblasts or lowering their collagen expression resulted in a worsened outcome and increased invasiveness 13,61. Similarly, the deletion of *PKN2* gene impaired the activation of mouse stellate cells upon TGFβ treatment and resulted in an increase in the inflammatory cancer-associated fibroblast phenotype, increased cancer cell invasiveness and a decreased survival in an animal study 42. This shows a dual, paradoxical role of myofibroblasts and ECM in pancreatic cancer: despite the known pro-cancer influence of these cells, they appear to simultaneously lower cancer invasiveness.

Besides desmoplasia, myofibroblasts were also noted to increase the level of immune-suppressing cells in the tumor stroma. In the tumor microenvironment, they were observed to be the main (although not the only) MFAP5-expressing cell type. The expression of this protein was correlated with worse prognosis, and knockdown experiments revealed that its expression is responsible for conferring increased migratory and invasive capabilities onto the pancreatic cancer cells, in addition to promoting the EMT in cancer cells and decreasing the tube-formation capability of HUVEC cells. These findings were recapitulated in a xenografted mouse model of pancreatic cancer. The proportion of immunosuppressive Treg and myeloid-derived Ly6G+ cells in the tumor was also decreased upon the knockdown of MFAP5 62. Moreover, the MFAP5-expressing fibroblasts were noted to secrete increased amount of hyaluronic acid and CXCL10, a cytokine which stimulated cancer cells to produce immunosuppressive PD-L1 62,63; knockdown of MFAP5 in fibroblasts was observed to sensitise the cancer to anti-PD-L1 immune therapy 62. Thus, MFAP5 may be understood as a main promoter of myofibroblasts' pro-cancer effects. Curiously, despite the observed inhibition of tumor angiogenesis by MFAP5-expressing myofibroblasts, depletion of myofibroblasts was also noted to decrease pancreatic tumor vascularity 13. It is possible that while the presence of MFAP5-expressing myofibroblasts inhibits the angiogenesis, there also exists a different, unidentified myofibroblast population, which supports angiogenesis, or that the ECM secretion is actually necessary for proper vasculature development. Curiously, even just decreasing the expression of collagen I in the myofibroblasts has resulted in an accumulation of myeloid-derived suppressor cells in the stroma 61, indicating a connection between myofibroblasts, the ECM and immune suppression in pancreatic cancer.

The presence of LRRC15 protein was also observed to denote an immune-suppressing myofibroblast subpopulation: TGFβR2 signalling activation in non-differentiated fibroblasts leads to the creation of LRRC15^+^ myofibroblasts, which form the most abundant fibroblast-related cell population within the pancreatic cancer microenvironment, as observed during an animal study. Depletion of that subset of myofibroblasts led to tumor growth rate inhibition due to an improvement in cytotoxic T CD8+ function. Moreover, LRRC15^+^ myofibroblasts-depleted tumors were more susceptible to anti-PDL-1 therapy 64. Thus, the presence of LRRC15 may denote a subset of myofibroblasts related to immune suppression. However, as the majority of cancer-associated myofibroblasts express this marker, it is possible that there are multiple LRRC15^+^ subpopulations, and depletion of myofibroblasts expressing this marker merely coincidentally results in anti-tumor effects. A single-cell transcriptomics study revealed that LRRC15^+^ cells represent the end stage of differentiation of a cell into the myofibroblast 30. MMP1-expressing myofibroblasts were also noted to be inducers of immune suppression in the pancreatic tumor, as they increased Treg migration upon coculture and were found, together with the LRRC15^+^ myofibroblasts, to be more numerous in tumors of patients which did not respond to immune therapy 30.

Hutton et al. have found a way to divide the population of pancreatic cancer-associated fibroblasts based on their expression of CD105 into a CD105^+^ immune-suppressive subset and a CD105^-^ immune-promoting fibroblasts. However, the expression of these markers was not confined to just myofibroblasts but to all pancreatic cancer-associated fibroblasts 65. Fibroblastic cells in PDAC interact with a number of other cell types besides the cancer cells and immune cells: pancreatic stellate cells may control angiogenesis in PDAC as they secrete both pro-angiogenic hepatocyte growth factor 66 and vascular endothelial growth factor 67 and increase the cancer cells' production of anti-angiogenic endostatin 67. They may also promote perineural invasion 68. However, none of these effects has been ascribed specifically to the myofibroblast subpopulation.

## Myofibroblasts as prognostic biomarkers

As myofibroblasts are identified in both high-grade precancerous lesions 14 and pancreatic cancer tumor 8,46, it appears that their emergence may either result in formation of pancreatic cancer, or at least be correlated with the disease's occurrence. Notably, myofibroblasts' influence on the prognosis of pancreatic cancer is controversial: it was once thought that a high αSMA-to-collagen ratio (and thus, a high proportion of myofibroblasts in the tumor's microenvironment) is associated with a worse outcome in pancreatic cancer patients 48, while a later study found that, for post-neoadjuvant therapy patients, a high proportion of myofibroblasts observed post-neoadjuvant chemotherapy is associated with a favourable outcome in patients treated with a neoadjuvant therapy, but not in patients who undergone resection without neoadjuvant therapy, for whom the low αSMA-to-collagen ratio was correlated with an increased survival 69. This demonstrates the possible significance of myofibroblasts for the efficiency of said therapy.

Yet another approach identified three stroma subtypes: αSMA-rich, FAP-rich and collagen-rich, with the last of these types being characterized by a significantly higher overall survival, while the αSMA-rich stroma was correlated with the lowest survival probability 49. This may further reinforce the notion that fibrous stroma is detrimental to pancreatic cancer progression, and that myofibroblasts are pro-tumor elements of pancreatic cancer stroma. Despite myofibroblasts being the main producers of collagen, surface of αSMA-stained areas did not correlate with stromal collagen content 49. As inflammatory cancer-associated fibroblasts are also known to express αSMA (though generally at a lower level than cancer-associated myofibroblasts) 32, it is possible that the αSMA-low collagen-high type of pancreatic cancer stroma is composed of highly ECM-secretory myofibroblasts, while the collagen-low αSMA-high type is composed predominantly of inflammatory cancer-associated fibroblasts 8 and CD90^-^ myofibroblasts 51. A high level of collagen may then restrict cancer progression 70, while a high level of inflammatory cancer-associated fibroblasts may enhance cancer progression by the secretion of pro-cancer cytokines 8.

The origin of myofibroblasts may also serve as a prognostic factor: pancreatic cancer-associated myofibroblasts of stellate cell origin are known to possess a unique ECM-related expression profile that increases ECM stiffness and the level of tumor the focal adhesion kinase signalling 20. That specific expression signature was correlated with a lowered patient survival 20. The possible mechanism through which the pancreatic stellate cells-derived myofibroblasts are associated with the disease outcome is unclear, but an observed post-depletion slight decrease in myeloid cell number within the tumor 20 may indicate that the activated pancreatic stellate cell-derived ECM serves as a niche for recruited immune cells, which then support the tumor. This is corroborated by a study by Wu et al., who observed a greater efficiency of drug delivery to the tumor after inducing pancreatic stellate cells quiescence 71. Overall, the result suggests that a high activity of pancreatic stellate cells-derived myofibroblasts worsens the outcome of the disease.

Based on the above, two myofibroblast-related prognostic biomarkers may be postulated. First, a low level of αSMA coupled with a high level of collagen in the pancreatic tumor, possibly indicating a predominance of ECM-producing myofibroblasts in the stroma, is indicative of a favourable prognosis. Second, a high number of myofibroblasts of stellate cell origin, possessing a specific ECM-secretory signature, is indicative of an unfavourable prognosis. Both of these potential biomarkers must be validated in clinical trials.

## Conclusions

Myofibroblasts are known to constitute a significant fraction of pancreatic cancer-associated fibroblast population 8. Several studies explain the origins of these cells, which includes transition from pancreatic stellate cells, development from bone marrow-derived progenitors, and activation of *in situ* fibroblasts. Importantly, both fibroblast and pancreatic stellate cell activation is caused by episodes of pancreatic damage (such as inflammation) and leads to them assuming a myofibroblast phenotype 18.

Mechanistically, the most important molecular pathway for fibroblast-to-myofibroblast transition is the TGFβ-NOX4-ATM kinase pathway, with Smoothened receptor signalling, PI3K/AKT/MAPK pathway signalling, mTOR activity and *PKN2* also described as relevant for myofibroblast phenotype attainment and maintenance 34,35,43. Tumor-associated macrophages also play a role in fibroblast-to-myofibroblast transition via the IL-33-ST2-CXCR2 axis 44.

While the aforementioned pathways present as attractive potential therapeutic targets, as decreasing the myofibroblast count could lead to a decreased fibrosis, and thus, to a higher drug penetration, it is important to note that attempting to decrease desmoplasia did not result in a better outcome in a clinical setting 72, and a total depletion of myofibroblasts worsens the disease's outcome 14,61,64. This may be due to a lack of mechanical barrier of collagen post-depletion, as it is known to hinder cancer cell invasion 70. Targeting only a subset of myofibroblasts expressing a specific marker instead could be a viable strategy. For example, depletion of LRRC15^+^ fibroblasts (expressed by the majority of them) was observed to result in a higher anti-tumor activity of CD8^+^ T cells and sensitize the tumor to immune therapy 64. Similarly, CD90^-^ myofibroblasts were noted to promote cancer cell metastasis, likely via secretion of pro-metastatic factors 49. These findings suggest that therapeutic modulation, rather than blanket elimination, may unlock the full clinical potential of targeting myofibroblasts.

Considerable research has been conducted aiming to determine contributions of different myofibroblast subpopulations to pancreatic adenocarcinoma: besides the above-mentioned LRRC15^+^ myofibroblast subpopulation, MFAP5^+^, MMP1^+^ and senescent myofibroblasts were implicated in immune suppression 30,50,62, while CD90^-^ and amphiregulin-secreting myofibroblasts were implicated in enhancing cancer cell proliferation or invasiveness 51. Stellate cell-derived pancreatic cancer-associated myofibroblasts are of a high-importance as their secretory phenotype was correlated with a worse outcome 20. **Table 1.** summarizes the described subpopulations of myofibroblasts in pancreatic adenocarcinoma. While these aforementioned subpopulation studies provide a possible clinical targets for PDAC management, a possible different approach, consisting of treating all cancer-associated fibroblasts as a spectrum and forming “functional units” together with the ECM instead of attempting to isolate specific subpopulations has recently been described by Francescone et al. 73.

Looking forward, the heterogeneity of myofibroblasts remains a central obstacle in pancreatic cancer. Utilizing high-throughput single-cell approaches will be essential for the full appreciation of distinct myofibroblast subtypes. By shifting the focus from elimination to modulation, future therapeutic strategies may improve drug delivery and overcome chemotherapeutic resistance by disrupting the pro-tumor functions of these cells.

## Figures and Tables

**Figure 1:**
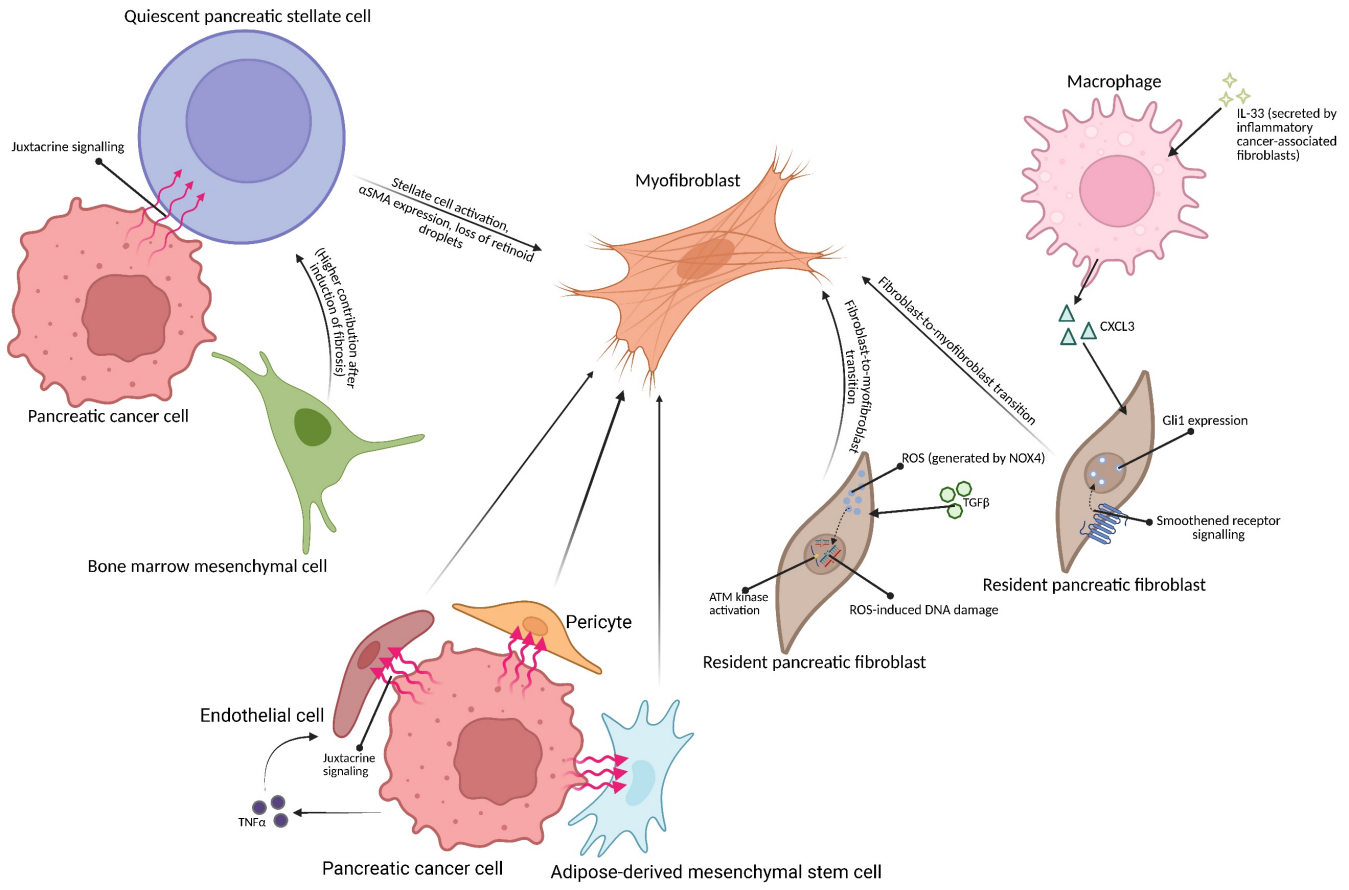
**Schematic representation of possible origins of myofibroblasts in pancreatic tumor microenvironment.** (Top left) Pancreatic cancer cell may activate the pancreatic stellate cell via juxtacrine means 8. The bone marrow-derived mesenchymal cell may differentiate into a quiescent pancreatic stellate cell 21, becoming activated by signalling from the cancer cell 8. (Right) Inflammatory cancer-associated fibroblast-secreted IL-33 causes macrophages in the tumor microenvironment to secrete CXCL3, causing fibroblast-to myofibroblast transition 44; TGFβ1 may also trigger fibroblast-to-myofibroblast transition in Gli-1-expressing fibroblasts 40. (Bottom) Endothelial cells, adipose-derived mesenchymal stem cells and pericytes may also be transformed into myofibroblasts due to signalling from pancreatic cancer cells 26,29,30. Created with BioRender.com

**Table 1:** Myofibroblast subpopulations in pancreatic adenocarcinoma and their functions.

Subpopulation	Function	Notes	Source
LRRC15^+^	Immune suppression	Possibly represents the most-differentiated myofibroblasts of pericyte origin	30,64
MFAP5^+^	Immune suppression, angiogenesis	-	62
MMP1^+^	Immune suppression	Enhance Treg motility, correlated with worse immune therapy outcome	30
Senescent	Immune suppression	Increase in number with disease progression	50
CD90^-^	Increase of cancer cell invasiveness	-	51
CD90^+^	ECM secretion	-	51
Autocrine amphiregulin-secreting	Stimulation of EMT, increase of cancer cell invasiveness	-	51
Stellate-cell derived	ECM secretion	Unique secretory signature correlated with worse outcome	20

## Abbreviations

αSMA: Alpha-smooth muscle actin
ATM: Ataxia telangiectasia mutated (kinase)
CD90: Cluster of Differentiation 90 (Thy-1 cell surface antigen)
CXCL3: C-X-C motif chemokine ligand 3
CXCL10: C-X-C motif chemokine ligand 10
DNMT1: DNA methyltransferase 1
ECM: Extracellular matrix
EGF: Epidermal growth factor
EGFR: Epidermal growth factor receptor
EMT: Epithelial-mesenchymal transition
FAP: Fibroblast activation protein
Gli-1: Glioma-associated oncogene homolog 1
HLA-II: Human leukocyte antigen class II
HUVEC: Human umbilical vein endothelial cells
IL-1: Interleukin 1
IL-6: Interleukin 6
IL-10: Interleukin 10
IL-33: Interleukin 33
KRAS: Kirsten rat sarcoma viral oncogene homolog
L1CAM: L1 cell adhesion molecule
LRRC15: Leucine-rich repeat containing 15
MFAP5: Microfibril-associated protein 5
MIA-PaCa-2: Human pancreatic cancer cell line
MYC: Myelocytomatosis oncogene
NOX4: NADPH oxidase 4
PDGF: Platelet-derived growth factor
PDGFR-α: Platelet-derived growth factor receptor alpha
PD-L1: Programmed death-ligand 1
PKN2: Protein kinase N2
PSC: Pancreatic stellate cell
ROS: Reactive oxygen species
SLUG: Zinc finger protein SNAI2
SOX9: SRY-box transcription factor 9
STAT1: Signal transducer and activator of transcription 1
ST2: Suppression of tumorigenicity 2 (IL-33 receptor)
TCA cycle: Tricarboxylic acid cycle
TGFβ: Transforming growth factor beta
TNF-α: Tumor necrosis factor alpha
Zeb1: Zinc finger E-box-binding homeobox 1
